# Supplementary material for: Targeting insulo-frontal pathway to reduce stress-evoked cognitive rigidity
Source: Nat Commun. 2026 Apr 27;17:5791. doi: 10.1038/s41467-026-72221-1 (PMC13332065; doi:10.1038/s41467-026-72221-1)
Supplement: Supplementary file 2 — Description of Additional Supplementary Files [file 41467_2026_72221_MOESM2_ESM.pdf]

Description of Additional Supplementary Files

File name: Supplementary Data 1

Description: An Excel file summarizing all the statistics presented in the paper
